# Designing a Multimer Allergen for Diagnosis and Immunotherapy of Dog Allergic Patients

**DOI:** 10.1371/journal.pone.0111041

**Published:** 2014-10-29

**Authors:** Ola B. Nilsson, Theresa Neimert-Andersson, Mattias Bronge, Jeanette Grundström, Ranjana Sarma, Hannes Uchtenhagen, Alexey Kikhney, Tatyana Sandalova, Erik Holmgren, Dmitri Svergun, Adnane Achour, Marianne van Hage, Hans Grönlund

**Affiliations:** 1 Department of Medicine, Clinical Immunology and Allergy Unit, Karolinska Institutet, Stockholm, Sweden; 2 Center for Biomembrane Research, Department of Biochemistry and Biophysics, Stockholm University, Stockholm, Sweden; 3 Department of Clinical Neuroscience, Therapeutic Immune Design Unit, Center for molecular Medicine (CMM), Karolinska Institutet, Stockholm, Sweden; 4 Science for Life Laboratory, Department of Medicine Solna, Karolinska Institutet, Stockholm, Sweden; 5 European Molecular Biology Laboratory (EMBL) - Hamburg Outstation, c/o DESY, Hamburg, Germany; Chang-Gung University, Taiwan

## Abstract

**Background:**

Dog dander extract used for diagnosis and allergen-specific immunotherapy is often of variable and of poor quality.

**Objective:**

To assemble four well-established dog allergen components into one recombinant folded protein for improved diagnosis and vaccination of allergy to dog.

**Methods:**

A linked molecule, comprising the four dog lipocalin allergens Can f 1, Can f 2, Can f 4 and Can f 6 was constructed. The tetrameric protein was structurally characterized by small angle X-ray scattering, and compared with each single recombinant lipocalin allergen or an equimolar mix of the four allergens by analytical size exclusion chromatography, circular dichroism, allergen-specific IgE in serum by ELISA and allergen-dependent capacity to activate basophils. The immunogenicity of the fusion protein was evaluated in immunized mice by assessing splenocyte proliferation and antibody production.

**Results:**

The linked tetrameric construct was produced as a soluble fusion protein, with the specific folds of the four individual allergens conserved. This multi-allergen molecule was significantly more efficient (p<0.001) than each single recombinant allergen in binding to dog-specific IgE, and the epitope spectrum was unaffected compared to an equimolar mix of the four allergens. Basophil degranulation revealed that the biologic activity of the linked molecule was retained. Immunization of mice with the linked construct induced comparable allergen-specific IgG responses with blocking capacity towards all included allergens and generated comparably low T-cell responses.

**Conclusion:**

We provide the first evidence for a linked recombinant molecule covering the major dog allergens for potential use in diagnostics and allergy vaccination of dog allergic patients.

## Introduction

Allergy to the domestic dog (*Canis familaris*) causes symptoms such as rhinitis, conjunctivitis and asthma. The pattern and complexity of sensitization to dog is distinctly different from that of cat. In cat dander, the major allergen Fel d 1 is recognized by up to ∼95% of all patients [1], whereas dog dander does not contain such a dominant allergen. The overall panel of dog allergens is so far incomplete for diagnostics of allergic patients, since some individuals only recognize minor allergen components [2], [3], [4], [5]. Four of the six presently known dog allergens, Can f 1, Can f 2, Can f 4 and Can f 6 [2], [4], [6], belong to the lipocalin protein family. These molecules share similar three-dimensional structures [3], [7] despite low sequence identity. Thus, no or low immunoglobulin cross-reactivity may be expected between these dog lipocalins [2], [8].

Allergen-specific immunotherapy (ASIT) is the only curative option for treatment of allergic diseases [9], [10], [11]. Diagnostics and immunotherapy of dog allergic patients is still based on conventional allergenic extracts, although such extracts have been shown to vary greatly in terms of allergenic contents [12]. In comparison to the cat, dog immunotherapy based on the use of natural extracts has been reported to have poor clinical efficacy [13], [14]. Furthermore, no recombinant vaccine for dog allergy has to our knowledge yet been presented.

Recombinant allergens can efficiently replace natural allergen extracts for the detailed characterization of IgE sensitization profiles, including component-resolved diagnostics [15]. Such constructs should retain important B- or T-cell epitopes, for potential improved ASIT [16], while limiting the problems associated with extract-based allergens and vaccination [17]. Previous clinical studies have demonstrated the efficiency of vaccination using single recombinant allergens from grass, birch and cat, which elicited protection to allergic disease [18], [19], [20]. However, no corresponding clinical trial with recombinant dog allergen has yet been performed.

In the present study, the first example of a designed recombinant molecule for improved diagnostics and vaccination of dog allergy is presented. A linked tetrameric molecule (designated Can f 1-2-4-6) that comprises four dog lipocalin allergens was generated, purified and biophysically characterized. The diagnostic value of the recombinant Can f 1-2-4-6 and respective single components was assessed by serum IgE responses and IgE inhibition by ELISA as well as by basophil activation test (BAT) using blood from dog-sensitized subjects. Finally, the efficacy of the Can f 1-2-4-6 construct as a vaccine candidate was investigated by monitoring proliferative and antibody responses in immunized mice. We conclude that the Can f 1-2-4-6 molecule may efficiently replace each individual dog lipocalin allergen included in the tetrameric construct, and might thus represent a valuable tool for diagnostics and treatment of dog allergic patients.

## Methods

### Human sera, dog allergens, construction and characterization of the linked molecule

Sera from 100 anonymous subjects with a positive reaction (≥0.35kU_A_/L) to dog dander extract were used for IgE testing. For more information on human sera and production of dog allergens, see the Methods section in File S1. The use of anonymous sera was approved by the regional ethics committee in Stockholm (EPN). The three patients selected for basophil activation test gave written informed consent and the study was approved by the regional ethics committee in Stockholm (EPN).

Detailed information on cloning, rational design, production and purification of the Can f 1-2-4-6 construct is contained in File S1. Briefly, the linked construct Can f 1-2-4-6 was assembled by PCR-based recombination of mature DNA sequences of Can f 1, Can f 2, Can f 4 and Can f 6 (See Figure S1 and Table S1 in File S1). The mature protein was synthesized in *E. coli* and purified using standard chromatographic methods.

### Small-Angle X-ray Scattering (SAXS) analysis

Structural characterization of the Can f 1-2-4-6 construct in solution was done based on small angle X-ray scattering (SAXS) data collected at the X33 camera of EMBL [21]. Primary data processing was done with the automated SAXS data analysis pipeline [22], *ab initio* shape reconstruction with the program GASBOR [23] and rigid body modelling with the program CORAL [24]. Additional information on SAXS data collection and analysis, biochemical characterization, preparation of equimolar amounts of allergens, standardization of lipopolysaccharide (LPS) content and circular dichroism (CD) analysis can be found in the Methods section within File S1.

### Serum IgE-responses and ELISA competition experiments

The IgE-reactivity of 100 dog-sensitized subjects to rCan f 1, rCan f 2, rCan f 4, rCan f 6, rCan f 1-2-4-6 and a mix of the included allergens was quantified as described [3]. ImmunoCAP binding characteristics of each allergen and the linked vaccine construct was assessed by competition ELISA as described [2]. Equimolar amounts to Can f 1 were used for all inhibition experiments.

### Allergen titrated basophil activation

Basophil activation test (BAT) was performed by analyzing allergen-specific basophil degranulation and monitoring the basophil marker CD203c and degranulation marker CD63 using flow cytometry [25]. Briefly, 10-fold serial dilutions of Can f 1, Can f 2, Can f 4, Can f 6, Can f 1-2-4-6 or a mix (10000 ng/ml to 0.01 ng/ml), medium (negative control), anti-human IgE and BCCR STCON (positive controls, kind gift from Thermo Scientific and Skafte medlab, Onsala, Sweden, respectively) was added to venous blood samples from three patients with physician diagnosed dog allergy, and detection of double positive CD203c+, CD63+ cells was carried out as described [26].

#### Immunization of mice

Six to eight week old female BALB/c mice were obtained from Charles River (Sulzfeld, Germany) and housed with food and water ad libitum. All animal experiments were approved by the Stockholm north ethical committee (Stockholms Norra Djurförsöksetiska nämnd). Groups of mice (n = 6) were immunized subcutaneously in the neck with 10 µg of more than 95% pure recombinant Can f 1 and equimolar amounts of the proteins Can f 2, Can f 4, Can f 6, Can f 1-2-4-6 or a mix. Two mice were immunized with natural (n)Can f 3 (control allergen) [27] and two with PBS (negative control). Each antigen was adsorbed to 1 mg aluminium hydroxide (Sigma-Aldrich, Steinheim, Germany). Injections were given on day 0, 14, 28 and the mice were sacrificed on day 35. Upon sacrifice, spleens were harvested for splenocyte culturing and blood collected by heart puncture.

### Investigation of splenocyte and immunoglobulin reactivity in female BALB/c mice

Splenocyte proliferation of cell suspensions prepared from spleens of immunized mice was essentially performed as described [28]. Cultured splenocytes (2×10^5^ cells/well) were stimulated in triplicates with 5 or 25 µg/ml of Can f 1 and equimolar amounts of the proteins Can f 2, Can f 4, Can f 6, Can f 1-2-4-6, mix, rFel d 4 (control allergen) [29], nCan f 3 (control allergen) or 5 µg/ml concanavalin A (positive control, Sigma-Aldrich) or left unstimulated. SI values were calculated, and the data was normalized as described [26].

Murine antibodies (IgG1, IgG2a and IgE) specific for rCan f 1, rCan f 2, rCan f 4 and rCan f 6 were analyzed in ELISA. In short, allergen-specific IgE measurement was evaluated using solid phase bound anti-mouse IgE followed by mouse antisera, biotinylated allergens, streptavidin-enzyme conjugate and finally substrate. Allergen-specific IgG antibodies were measured by solid-phase bound allergen, followed by mouse antiserum, sub-class specific secondary antibody enzyme conjugate and substrate as described [26]. Sera were diluted 1∶20 for IgE, 1∶5000 for IgG1 and 1∶250 for IgG2a.

The ability of the mouse antibodies to block IgE binding of serum from 6 subjects sensitized to each of the four lipocalin allergens was determined by ELISA [30]. Plates were coated with 5 µg/ml of either rCan f 1, rCan f 2, rCan f 4 or rCan f 6. Serum pools from mice immunized either with rCan f 1, rCan f 2, rCan f 4, Can f 6, the mix, Can f 1-2-4-6 or serum from naïve mice were diluted 1∶10, added to the wells and incubated for 2 hours at RT. Pooled human sera from dog sensitized subjects diluted to 0.3 kU_A_/L were added to wells for 2 hours, followed by detection of IgE responses as described.

### Statistical analysis

For information on statistical analysis, see File S1.

## Results

### Construction and biochemical characterization

**Figure 1:**
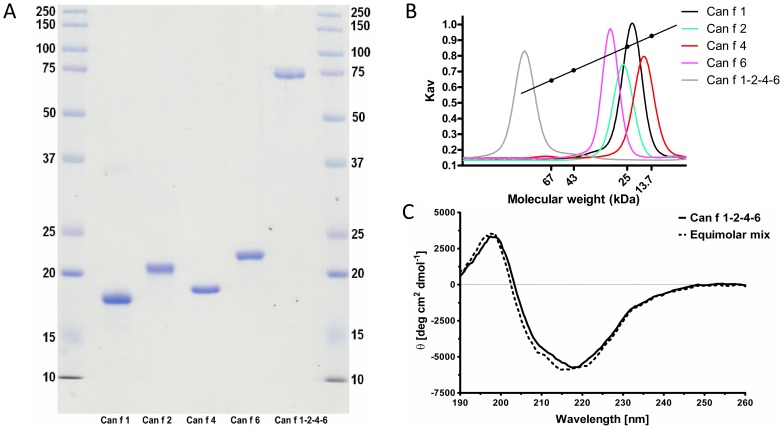
Biochemical analysis of the linked molecule. (A) SDS-PAGE of Can f 1-2-4-6 or the single recombinant lipocalins visualized by coomassie staining under reducing conditions. MW markers (lane 1,7, MW (kDa)), rCan f 1 (lane 2), rCan f 2 (lane 3), rCan f 4 (lane 4), rCan f 6 (lane 5) and Can f 1-2-4-6 (lane 6). (B) Analytical size exclusion chromatography of rCan f 1-2-4-6 and the single lipocalin allergens. Molecular weight markers, bovine serum albumin (67 kDa), ovalbumin (43 kDa), chymotrypsinogen A (25 kDa) and Ribonuclease A (13.7 kDa). (C) Far-UV CD analysis of Can f 1-2-4-6 or an equimolar mix of the single recombinant allergens Can f 1, Can f 2, Can f 4 and Can f 6. The spectra are expressed as mean residue ellipticities (θ) at a given wavelength.

The linked tetrameric molecule consisting of the lipocalins Can f 1, Can f 2, Can f 4 and Can f 6 was assembled by overlapping PCR recombination, introducing three flexible linkers each composed of the same stretch of amino acid residues Gly-Ser-Gly-Ser between each individual allergen. Excellent initial yields (10 mg/L culture media) of soluble recombinant fusion protein were obtained and a homogenous (Fig. 1a) and stable monomeric fusion protein of approximately 75 kDa was produced after Ni^2+^ affinity chromatography, His-tag removal, ion exchange and size exclusion chromatography (SEC). The Can f 1-2-4-6 molecule eluted as a symmetrical peak, with an approximate molecular weight of ∼90-100 kDa (Fig. 1b). CD analysis was used to assess the three-dimensional fold of Can f 1-2-4-6 compared to an equimolar mix of the four corresponding lipocalin allergens. The resulting spectra were highly similar, indicating that the linkers did not disturb the fold of each allergen within the tetramer (Fig. 1c).

### Structural analysis of Can f 1-2-4-6

**Figure 2:**
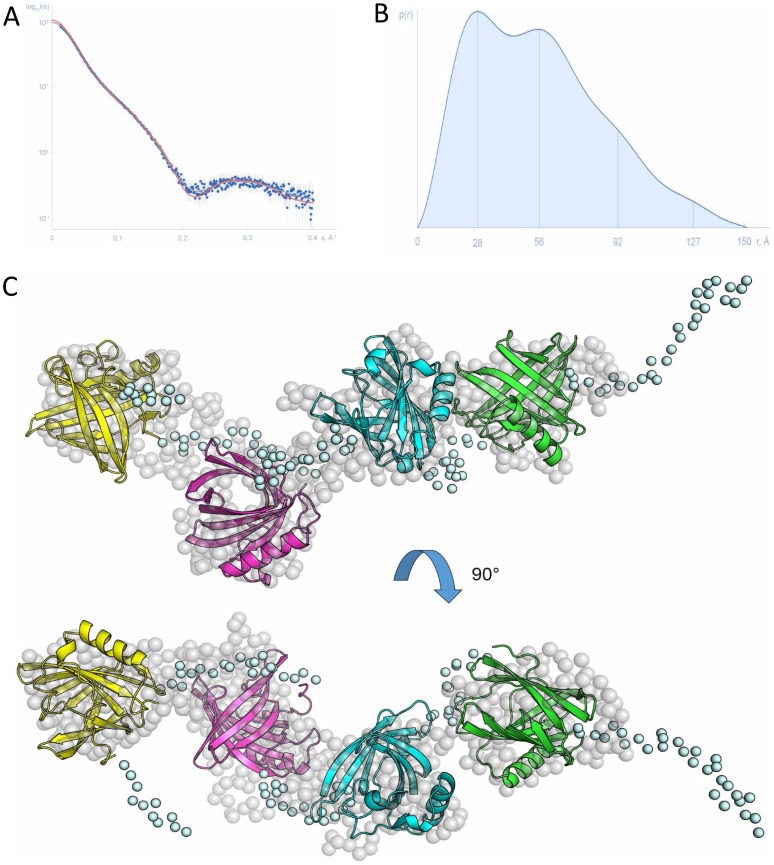
Can f 1-2-4-6 structure based on solution small angle X-ray scattering data. (A) Experimental SAXS data from Can f 1-2-4-6 in solution (blue dots with error bars), computed fits from the CORAL (red line) and the GASBOR (grey line) models. (B) Respective pair distance distribution function. Maxima marked by vertical lines correspond to distances between the individual domains. (C) The rigid body model of Can f 1-2-4-6 reconstructed by the program CORAL resembles beads on a thread with each lipocalin forming a bead (cartoon representation) interspaced by extended linkers (light blue spheres). This model is in a good agreement with the *ab initio* GASBOR model (dummy residues represented by grey spheres).

The overall three-dimensional structure of Can f 1-2-4-6 was investigated using SAXS in order to provide a structural basis for the high immunogenicity and diagnostic potential of the tetrameric construct compared to the previously determined crystal structures of individual dog lipocalin allergens [3]. The processed SAXS pattern from Can f 1-2-4-6 is displayed in Fig. 2a. The molecular weights estimated from the forward scattering I(0) and from the Porod volume were 77±8 kDa and 75±8 kDa, respectively (76.4 kDa expected for a four-domain construct). The radius of gyration R_g_ obtained from the Guinier approximation and the maximum intraparticle distance D_max_ were 42.1±3.2 Å and 152±15 Å, respectively, which indicates an extended structure in solution. The overall parameters are summarized in Table S2 in File S1. The computed distance distribution function p(r) displays maxima at distances 28 Å, 56 Å and 92 Å (Fig. 2b) that is characteristic of elongated particles with periodic domain arrangements [31]. The reconstructed models of Can f 1-2-4-6 resemble beads on a thread with each lipocalin forming a bead interspaced by a Ser/Gly linker (Fig. 2c). Importantly, none of the lipocalins composing Can f 1-2-4-6 interacts with any of its neighbours. Thus, the spatial arrangement of the four lipocalin allergens within the tetramerical vaccine most probably allows the surface of each lipocalin to be readily available for interaction with the IgE antibody repertoire from patients.

### IgE-binding properties

**Figure 3:**
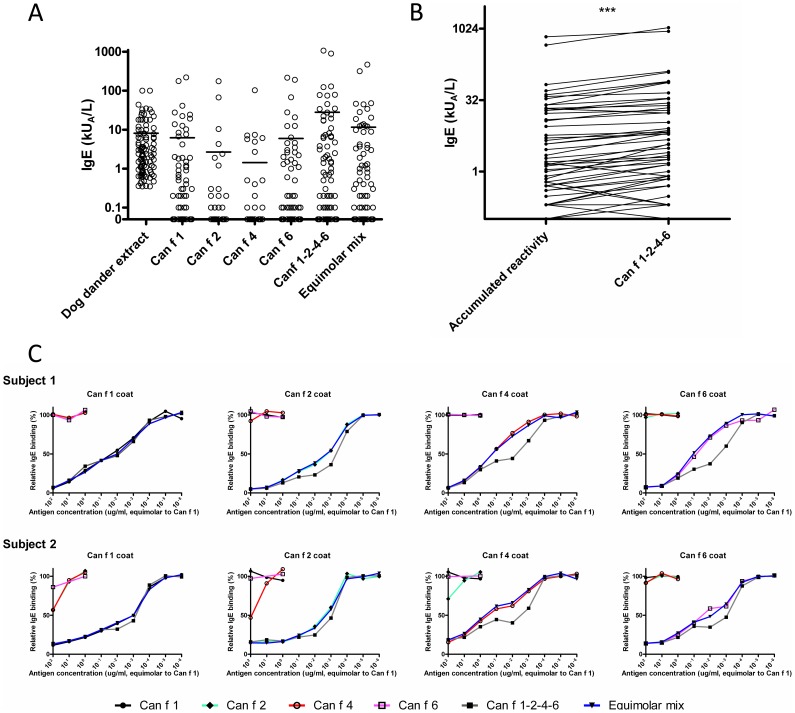
IgE-binding properties of the linked construct. (A) IgE-reactivity of 100 consecutive dog-sensitized subjects (e5, >0.35kU_A_/L, ImmunoCAP, Thermo Fisher scientific) towards dog extract, Can f 1-2-4-6, the single allergens rCan f 1, rCan f 2, rCan f 4 and rCan f 6, or an equimolar mix of the same allergens. Bars denote mean values. Data are presented as adjusted kU_A_/L. (B) Pair-wise comparison of accumulated IgE-reactivities to Can f 1, Can f 2, Can f 4 and Can f 6 among positive subjects with reactivity to Can f 1-2-4-6. *** p<0.001 (Wilcoxon matched pairs test) (C) Competitive inhibition ELISA with sera from subjects sensitized to rCan f 1, rCan f 2, rCan f 4 and rCan f 6. After pre-incubation of sera with 10-fold dilutions of equimolar amounts of each recombinant allergen, mix or Can f 1-2-4-6, IgE-responses were measured. Inhibition was calculated as response in percentage (%) of response to the coated antigen after allergen addition (y-axis). Subject 1 and 2 respectively correspond to subject 8 and 21, Table S3 in File S1.

IgE-binding frequencies in sera from dog sensitized subjects (n = 100) to the allergens were 45%, 16%, 14%, 39%, 59% and 53% for rCan f 1, rCan f 2, rCan f 4, rCan f 6, Can f 1-2-4-6 and the equimolar mix, respectively (Fig. 3a, Table S3 in File S1). In most cases the IgE-levels to the linked construct were significantly higher compared to the accumulated reactivity of the four individual lipocalin allergens or the equimolar mix (p<0.001) (Fig. 3b). Occasionally, the Can f 1-2-4-6 construct also elicited an IgE-response, where all four individual allergens were negative. We found no case where Can f 1-2-4-6 was negative and a single allergen positive.

The IgE-binding of Can f 1-2-4-6 was analysed using antigen competition ELISA. Neglible cross-reactivity was observed between the four lipocalin allergens (Fig. 3c). Also, no major difference in inhibiting capacity was observed between the equimolar mix and each individual allergen.

### Biologic activity by basophil activation test

**Figure 4:**
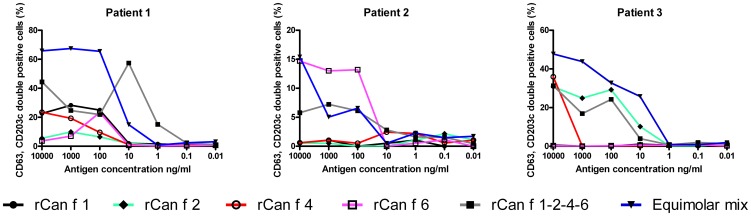
Biologic responses to Can f 1-2-4-6 among dog allergic patients. Allergen-specific basophil degranulation monitored by addition of serial dilutions of rCan f 1 or equimolar amounts of either rCan f 2, rCan f 4, rCan f 6, an equimolar mix or Can f 1-2-4-6 (x-axis) to whole blood from three dog allergic patients. Positivity was defined by analysis of CD63 and CD203c double positive cells (y-axis) using flow cytometry.

The allergenic activity of Can f 1-2-4-6 was compared to each individual lipocalin allergen or to the equimolar mix of allergens by assessing their ability to upregulate degranulation markers on basophils from dog allergic patients using BAT. An increase in sensitivity (x-axis) was observed in basophils from patient 1 using Can f 1-2-4-6 compared to the equimolar mix of all four allergens (Fig. 4). However, it should be noted that the reactivity (y-axis) of the equimolar mix was higher compared to the linked tetramerical molecule. Importantly, the individual allergens exhibited both lower sensitivity and reactivity compared to Can f 1-2-4-6 and to the equimolar mix of allergens. Two patients, sensitized to Can f 6 (patient 2) and Can f 2 (patient 3) by ELISA, exhibited similar responses to Can f 1-2-4-6, the equimolar mix and the individual allergens, and no increase in sensitivity was detected with Can f 1-2-4-6. Additionally, patient 3 reacted to Can f 4, which could not be detected by ELISA, highlighting the sensitivity of BAT.

### Proliferation of splenocytes from immunized mice

**Figure 5:**
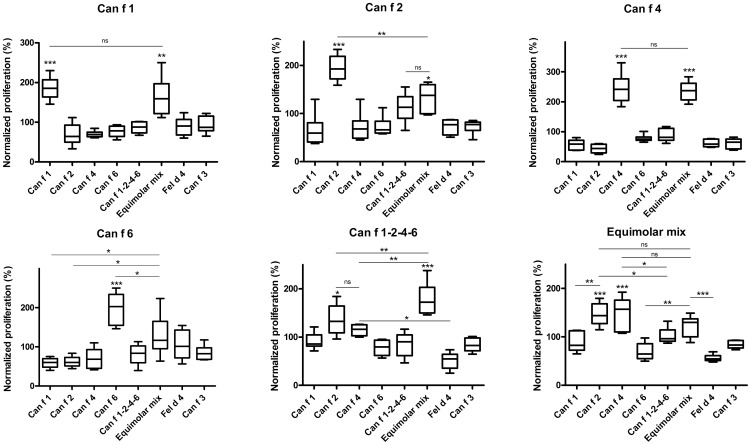
Splenocyte proliferation assay in immunized mice. Proliferative responses after stimulation of splenocytes from immunized mice (immunization allergen denoted as individual graph heading), stimulated with equimolar amounts of rCan f 1, rCan f 2, rCan f 4, rCan f 6, an equimolar mix or Can f 1-2-4-6 (x-axis) and measured by [3H] thymidine incorporation (y-axis). Boxes with median values and horizontal bars denote 50% of values and 1 standard deviation respectively. * p<0.05, ** p<0.01, *** p<0.001 and ns (non significant) p>0.05, analyzed with repeated measurements ANOVA with Tukey's multiple comparison test

The proliferative response in stimulated splenocytes from immunized mice was investigated by [3H]-thymidine-incorporation. The result of the two allergen concentrations was similar, here displayed by 5 µg/ml. A strong proliferation was observed upon stimulation of splenocytes from mice immunized with either rCan f 1, rCan f 2, rCan f 4 or rCan f 6, compared to the control allergens rFel d 4 and nCan f 3 (p<0.001) (Fig. 5). Similar amplitude was exhibited by the equimolar allergen mix on splenocytes from mice immunized with rCan f 1 or rCan f 4, whereas rCan f 2 and rCan f 6 displayed significantly lower proliferation compared to each individual recombinant allergen. Interestingly, no significant proliferation was observed upon exposure to Can f 1-2-4-6 in any of the treated groups, including mice immunized with Can f 1-2-4-6. Conversely, splenocytes from mice immunized with Can f 1-2-4-6 proliferated significantly when stimulated with either rCan f 2, rCan f 4 or the allergen mix. Furthermore, mice immunized with the allergen mix displayed strong proliferative responses upon stimulation with rCan f 2, rCan f 4 or the mix.

### Analysis of antibody responses in sera from immunized mice

**Figure 6:**
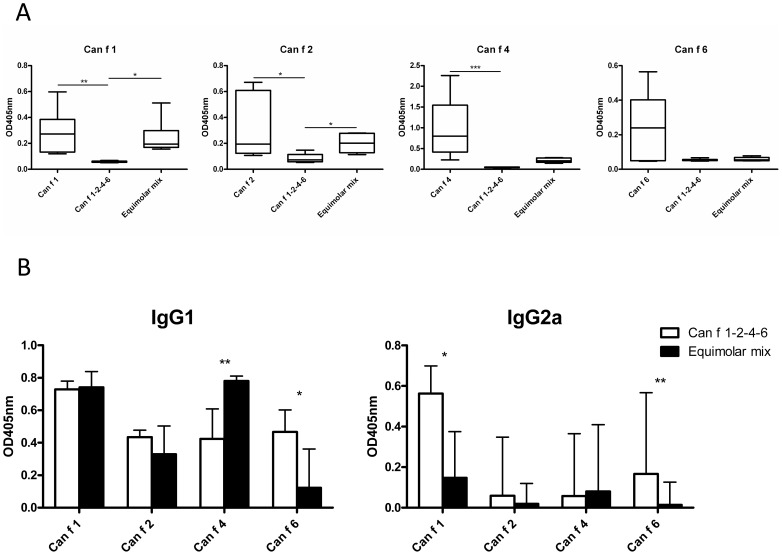
Antibody responses after immunization with allergens. (A) quantification of IgE-responses in mice (n = 6) immunized with each rCan f 1, rCan f 2, rCan f 4, rCan f 6, an equimolar mix or Can f 1-2-4-6 (x-axis in graphs) to plates coated with anti-mouse IgE, mouse antiserum and biotinylated allergen (graph heading) by sandwich ELISA. (B) Comparison of IgG1 and IgG2a-antibodies to each lipocalin induced by solid phase Can f 1-2-4-6 or a mix. Immunoglobulin responses are presented as OD405 nm, y-axis. Boxes with median values and horizontal bars denote 50% of values and 1 standard deviation respectively. * p<0.05, ** p<0.01, *** p<0.001, analyzed with Kruskal-Wallis with Dunn's multiple comparison test (A, B, C) and Mann Whitney test (D).

Mice immunized with the single recombinant allergens rCan f 1, rCan f 2 and rCan f 4 displayed significantly higher allergen-specific IgE responses compared to mice immunized with Can f 1-2-4-6 (Fig 6a). Furthermore, mice immunized with the allergen mix had significantly higher IgE responses to Can f 1 and Can f 2, compared to mice immunized with Can f 1-2-4-6 (Fig 6a). In addition, the allergen mix displayed significantly higher IgE responses to itself compared to the multimer, (Fig. S2a in File S1).

Upon analysing IgG responses, we noted that allergen-specific IgG1 was induced after immunization with all single recombinant allergens (Fig. S2b in File S1). Except for immunization with rCan f 1, IgG2a responses were low among animals treated with single recombinant allergens (Fig. S2b in File S1). Mice immunized with Can f 1-2-4-6 displayed comparable IgG1 levels to all four allergens, and only rCan f 1 levels were significantly higher (Fig. 6b). In contrast, responses in mice immunized with the allergen mix varied, with significantly higher levels to rCan f 1 and rCan f 4 compared to rCan f 6 (p<0.01), and a trend towards higher levels compared to rCan f 2 (p<0.2). Finally, mice immunized with Can f 1-2-4-6 had significantly higher anti-rCan f 6 levels compared to mice immunized with the allergen mix. The opposite was observed for rCan f 4, where the mix exhibited higher levels. The linked molecule also induced significantly higher IgG2a levels to rCan f 1 and rCan f 6 than the mix (Fig. 6b).

### Assesment of IgE-blocking effect of the mouse sera

**Figure 7:**
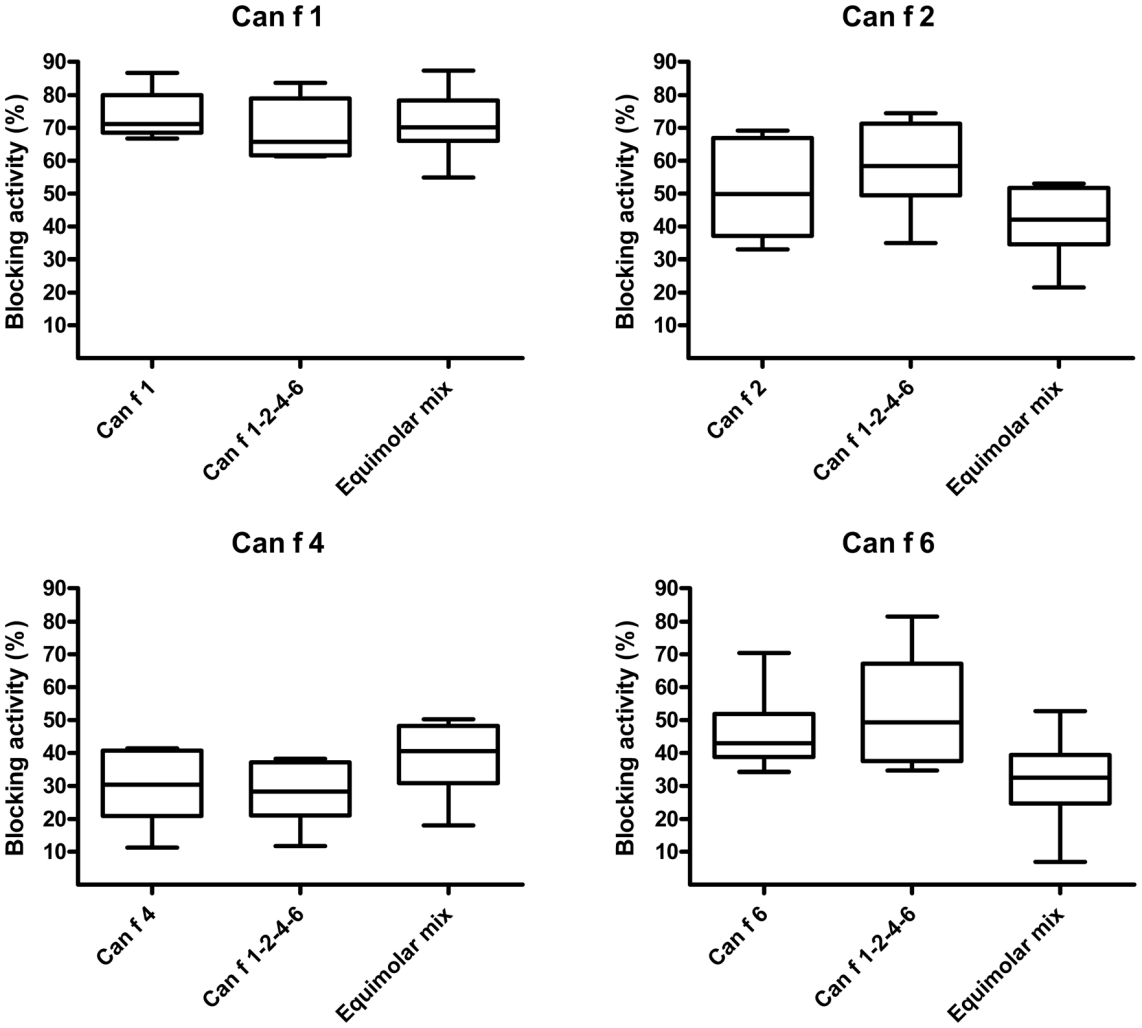
Human serum IgE blocking capability of IgG antibodies induced by the linked construct. Pooled sera from mice (n = 6) immunized with each of rCan f 1, rCan f 2, rCan f 4, rCan f 6, Can f 1-2-4-6 or an equimolar mix was preincubated in wells coated with either rCan f 1, rCan f 2, rCan f 4 or rCan f 6. Sera from multi-sensitized subjects (n = 6) was added to the wells and IgE-responses recorded. The percentage of IgE inhibition is displayed on the y-axis. Median values are indicated in the boxes. The boxes and horizontal bars denote 50% of values and 1 standard deviation respectively.

The sera from mice immunized with either the single recombinant allergens, the allergen mix or the linked Can f 1-2-4-6 construct did not significantly vary in the capacity to block binding of human allergen-specific IgE to the respectively allergen (Fig. 7). No blocking effect was displayed by serum from naïve mice.

## Discussion

A great variability in the quality of commercial dog dander extracts has been recently reported [12] and additionally, ASIT with these complex allergenic extracts also show very poor clinical efficacy [12], [13]. A way to control the natural variability of extracts is to use recombinant techniques to devise suitable vaccine combinations. A long list of different approaches has been reported e.g. hypoallergen variants, mix of allergens or mosaic multi allergens [32]. Nevertheless, it should be noticed that the vast majority of ASIT is extract based, containing allergens presumably in a natural conformation. In this study we propose a single, soluble and structurally examined recombinant allergen molecule comprising four linked important dog allergens as a viable alternative for improved diagnostics and ASIT of dog allergic patients.

Biochemical and structural analysis demonstrated that the four important dog allergens can be linked and produced in a pure, well-folded and soluble tetrameric Can f 1-2-4-6 molecule. CD spectroscopical analysis confirmed nearly identical compositions of α-helices and β-sheets between Can f 1-2-4-6 and an equimolar mix of the four allergens. The overall structure determined using SAXS revealed that the four lipocalins were independently positioned. The intrinsic flexibility of the linkers in between each lipocalin probably allows for additional freedom of rotation for each allergen domain. The surfaces of the four allergens were readily accessible to the solvent, allowing antibody binding.

Competition ELISA assays established that the linked tetrameric allergen covered the entire IgE-epitope spectrum elicited by each lipocalin allergen included in the construct. IgE to the tetrameric molecule was detected in all subjects positive to at least one of the lipocalin allergens, but also in a few individuals that were negative using each respective allergen. Furthermore, we analysed IgE antibodies in serum from 100 dog sensitized individuals to evaluate the IgE profile of the different lipocalins and the possible prevalence of patients eligible for immunotherapy. Among 39% of subjects positive to any lipocalin a greater IgE response to Can f 1-2-4-6 was noted compared to the extract, which is a likely result after suboptimal exposure of extract-derived allergens to patient IgE. It is thus conceivable that dog-sensitized patients with a predominance of IgE to lipocalins would be suitable candidates for efficacious ASIT using the Can f 1-2-4-6 construct. Interestingly, some patients, e.g. 3, 8, 14, 21 etc showed high or very high responses to the multimer compared to the single lipocalin components. This is speculatively a result of increased avidity of cross-reactive allergen-specific IgE antibodies due to the proximity of lipocalins in the linked multimer. The clinical relevance of this observation remains to be validated.

The biological activity of Can f 1-2-4-6 was compared for each individual allergen or the mix of allergens by BAT. Multi lipocalin sensitized patients (i.e. patient 1, fig. 4) seemed to be more sensitive to Can f 1-2-4-6 compared to the mix of allergens or each allergen individually, perhaps due to an enhanced local concentration of cross-linked IgE antibodies on the basophils caused by the tetrameric construct. As displayed by BAT, allergens with a natural fold have a potential to induce immediate allergic reactions. The multimer contains several linked allergens, of which each carries a propensity to induce mediator release. As with any allergen, caution must thus be exerted using the multimer for ASIT. Basophils from patients sensitized to just one of the lipocalin allergens reacted similarly or more vigorously to the mix or to the single allergens compared to Can f 1-2-4-6. Thus the sensitivity and reactivity of the allergens in the BAT seemed dependent foremost on the great variation in sensitization profile of the patient, Table S3 in File S1.

Several mechanisms for successful ASIT have been proposed, including the formation of blocking IgG antibodies that compete with IgE for binding to surface epitopes on the allergens [33]. The immunogenic potential of Can f 1-2-4-6 as a vaccine candidate was therefore investigated in mice. We found that the individual recombinant allergens induced potent T-cell responses. The single rCan f 2 and rCan f 6, exhibited significantly higher reactivity than the allergen mix, suggesting interference of the T cell peptide presentation by lipocalin-derived epitopes other than the stimulus. The linked vaccine candidate did not induce higher proliferative responses in any of the cultures. Furthermore, the proliferation of splenocytes from mice immunized with Can f 1-2-4-6 was always significantly stronger with rCan f 2, rCan f 4 and the allergen mix, compared to the Can f 1-2-4-6 tetramer, which might reflect a different route of uptake. It was also evident that rCan f 2 and rCan f 4 dominated the proliferative responses in cultures from mice immunized with the mix. Individual LPS levels (File S1) did not appear to affect the result of the splenocyte proliferation assay. These results indicate that the linked construct seems to modulate T cell responses for moderate allergen recognition, without inducing strong proliferative responses. ASIT with recombinant allergens is clinically effective, but might be limited by late phase reactions (LPR), caused by uncontrolled T cell responses [33], [34]. Consequently, our dog allergen molecule may be safer for use in ASIT, by carrying a lower risk of eliciting LPR.

We also analysed antibody responses in mice. Single recombinant allergens induced significantly higher allergen-specific IgE levels than mice immunized with the linked molecule. In addition, mice immunized with the mix displayed significantly higher IgE responses to itself, rCan f 1 and rCan f 2 compared to Can f 1-2-4-6. Consequently, vaccination with the linked construct may also carry a lower risk of inducing novel IgE responses, which would be an attractive feature in ASIT.

Immunization with the linked construct induced high titers of allergen-specific IgG1 to all individual allergens. Interestingly, compared to the mix, immunization with the linked molecule resulted in high and even antibody responses. In particular, anti-Can f 6 IgG levels induced by the mix were low and conversely pair-wise analysis confirmed that Can f 1-2-4-6 induced higher IgG1-levels to rCan f 6, while the mix induced stronger responses to rCan f 4. The responses of Can f 6 compared to the other dog lipocalins may be a result of high shared amino acid sequence identity, about 60%, between homologous proteins in other species, e.g. allergens in mouse Mus m 1, horse Equ c 1 and cat Fel d 4. This comparably high sequence identity would tentatively render the Can f 6 allergen less immunogenic. We suggest that Can f 6 given as single molecules, either by itself or as a component in the mix, induces relatively lower antibody titres. Conversely, when physically linked to more immunogenic proteins, a by-stander effect would tentatively enhance the immunogenicity. We argued that the overall high allergen-specific IgG levels of the linked construct are preferential over a variable high and low antibody responses in allergen mixes. Thus, the concept of allergen linkage appears to boost immunoglobulin responses to otherwise weak antigens. In addition, Can f 1-2-4-6 also induced significantly stronger IgG2a-responses towards rCan f 1 and rCan f 6, compared to the mix, indicative of a Th1 cellular response, which may be a beneficial for successful ASIT [34]. Notably, high antibody responses were obtained with the linked molecule despite low splenocyte proliferation. This indicates that a strong T cell response is not a necessity for the induction of blocking IgG responses, which might favour a lower risk of inducing LPR.

Finally, we investigated whether the obtained allergen-specific antibodies could block serum IgE binding to each respective recombinant allergen among six sensitized subjects. Importantly, we did not detect any differences in blocking activity between the single allergens, the mix or the linked construct indicating maintained allergen structures and epitopes which should be advantageous for successful ASIT.

Herein this study we present a well-characterized recombinant allergen for diagnostics and improved ASIT of dog allergic patients. The multimeric allergen construct comprises several desirable features including an increased diagnostic sensitivity aimed at improved selection of dog sensitized subjects and with respect to allergy vaccination, an ability of inducing high concentrations of dog-allergen specific blocking IgG antibodies and an improved safety profile as it displayed a significantly lower T cell activation capacity and a reduced IgE production compared to the mix of dog allergens.

## Supporting Information

File S1 **This file contains supporting information, including Figure S1, Figure S2, and Table S1- Table S3.** Figure S1, Cloning of the Can f 1-2-4-6 construct. Figure S2, Antibody responses after immunization with allergens. Table S1, List of oligonucleotides used for construction of the Can f 1-2-4-6 construct. Table S2, SAXS data collection and derived parameters. Table S3, Dog sensitized subjects and IgE-levels against tested allergens.
(DOC)
