# Supplementary material for: Designing a Multimer Allergen for Diagnosis and Immunotherapy of Dog Allergic Patients
Source: PLoS One. 2014 Oct 29;9(10):e111041. doi: 10.1371/journal.pone.0111041 (PMC4212987; doi:10.1371/journal.pone.0111041)
Supplement: File S1 — This file contains supporting information, including Figure S1, Figure S2, and Table S1- Table S3. Figure S1, Cloning of the Can f 1-2-4-6 construct. Figure S2, Antibody responses after immunization with allergens. Table S1, List of oligonucleotides used for construction of the Can f 1-2-4-6 construct. Table S2, SAXS data collection and derived parameters. Table S3, Dog sensitized subjects and IgE-levels against tested allergens. (DOC) [file pone.0111041.s001.doc]

### Designing a multimer allergen for diagnosis and immunotherapy of dog allergic patients

**Ola B. Nilsson^1,2^, Theresa Neimert-Andersson^3^, Mattias Bronge^3^, Jeanette Grundström^1^, Ranjana Sarma^4^, Hannes Uchtenhagen^4^, Alexey Kikhney^5^, Tatyana Sandalova^4^, Erik Holmgren^1,3^, Dmitri Svergun^5^, Adnane Achour^4^, Marianne van Hage^1^ and Hans Grönlund^3^ *Stockholm, Sweden and Hamburg, Germany***

**1.** Department of Medicine, Clinical Immunology and Allergy Unit, Karolinska Institutet, Stockholm, Sweden

**2.** Center for Biomembrane Research, Department of Biochemistry and Biophysics, Stockholm University, Stockholm, Sweden

**3.** Department of Clinical Neuroscience, Therapeutic Immune Design Unit, Center for molecular Medicine (CMM), Karolinska Institutet, Stockholm, Sweden

**4.** Science for Life Laboratory, Department of Medicine Solna, Karolinska Institutet, Stockholm, Sweden

**5.** European Molecular Biology Laboratory (EMBL) - Hamburg Outstation, c/o DESY, Notkestrasse 85, 22603 Hamburg, Germany

**Corresponding author: Hans Grönlund, Department of Clinical Neuroscience, Therapeutic Immune Design Unit, Center for molecular Medicine (CMM), Karolinska Institutet, Stockholm, Sweden. E-mail: hans.gronlund@ki.se**

### Supporting information

### Methods

*Subjects*

Sera from 100 anonymous consecutive subjects with IgE reactivity ≥ 0.35 kUA/L (ImmunoCAP™, Phadia, Uppsala, Sweden) to dog dander extract (e5) were collected from the routine diagnostics lab (Department of Clinical Immunology and Transfusion Medicine at the Karolinska University Hospital, Solna). The use of anonymous sera was approved by the regional ethics committee in Stockholm (EPN).

Three patients with physician diagnosed allergy to dog, and a positive ELISA test to at least 1 recombinant dog allergen , were selected for basophil activation test. The patients gave written informed consent. The study was approved by the regional ethics committee in Stockholm.

*Cloning, production and purification of recombinant allergens*

Recombinant (r) Can f 1, rCan f 2 and rCan f 6 were produced and purified as previously described . rCan f 4 was constructed, produced and purified as described for rCan f 6. Briefly, the coding sequence was purchased from Geneart (Regensburg, Germany) after removal of the signal peptide from the complete open reading frame (NP_001177855.1), using SignalP (<http://www.cbs.dtu.dk/services/SignalP/>). The fragment was subcloned into the vector pET20b (Novagen, EMD Chemicals Inc., Darmstadt, Germany) resulting in a 6x HIS-tagged protein, and then produced and purified as described .

*Design, cloning, production and purification of the linked molecule*

A modified version of the pET28 vector (Novagen) was constructed for purposes of ligase independent cloning (LIC) of the Can f 1-2-4-6 construct (Figure S1a). The plasmid was constructed with green fluorescent protein (GFP) used as monitor protein for detection of background colonies, together with an enterokinase cleavage site (EK) for removal of the N-terminal 6x-HIS tag (Fig. S1a). The gene of interest is directionally subcloned using two LIC-sites by the nicking enzyme Nt.BbvCI (New England Biolabs, Ipswich, MA, USA).

The linked construct Can f 1-2-4-6 was assembled by PCR-based recombination of mature DNA sequences of each separate antigen (Figure S1b). The individual allergens were amplified in a first step using overlapping oligonucleotides with overhangs designed to create Gly-Ser-Gly-Ser linkers between each antigen (Figure S1b, Table. S1); primer 1,2 for Can f 1, primer 3,4 for Can f 2, primer 5,6 for Can f 4 and primer 7,8 for Can f 6. 5’ region ends of Can f 1-2-4-6 Forward and Reverse primers respectively, (primers 1 and 8), were designed to obtain unique cloning sites by adding the sequences 5’-TCAGCAAGGGCTGAGG→ and 5’-TCAGCGGAAGCTGAGG→ respectively (→ indicating additional bases specific for Can f 1 and Can f 6 respectively), for LIC into pET28-H6-EK-sgGFP. A site for EK cleavage (gatgatgacgataaa, encoding Asp-Asp-Asp-Asp-Lys) of the His-tag was added 5’ to the Can f 1 using primer 1, and a stop codon (TAATAA) was included in the Can f 6 fragment using primer 8.

After amplification of the individual fragments, the full construct was fused using primer 1 and 8, annealing the individual fragments to the complete linked molecule Can f 1-2-4-6, containing the four allergens in order Can f 1 – Can f 2 – Can f 4 – Can f 6.

The fragment was ligated into pET28-H6-EK-sgGFP, previously opened in the sgGFP gene with SpeI to reduce background colonies. Nicking and annealing was performed in a volume of 20 µl mix, containing 2 ul 10xNEB4 buffer, 20 ng vector DNA and 100 ng PCR fragment, after adding 5 Units of Nt.BbvCI and incubation 1h at 37°C, 10 minutes at 80°C, and 10 minutes at 30°C. 2.5 µl of this material was used for transformation of 25 µl chemically competent cells (TOP10, Life Technologies, Paisley, UK). Colonies were screened for positive inserts of correct size with PCR using primer 9 and 10 (T7 Promoter and Terminator sequencing primers), and the final construct was fully sequenced using primer 2, 3, 7, 8, 9 and 10 (Table S1).

The vector DNA containing the final linked vaccine construct was prepared (Plasmid Miniprep Spin Kit, Genomed, Löhne, Germany) and transformed into E. coli BL21 (DE3) cells. Cells were grown in Super broth medium with 50 mg/L kanamycin to OD550 of 0.7, and induction of protein expression was done by addition of isopropyl-b-thiogalactopyranoside (IPTG) to a final concentration of 0.4 mmol/L. The cells were grown for 4 hours, spun down (18  600g), dissolved in PBS and sonicated (Soniprep 150 ultrasonic disintegrator, Sanyo Gallenkamp, Uxbridge, UK) by 15 sec (10 ma) bursts on ice and centrifuged at 18600 g for 10 min where a soluble fraction was obtained. The soluble fraction was loaded on a 5-ml Ni2+-HiTrap IMAC column (GE Healthcare, Uppsala, Sweden), and eluted by adding 5 mM Tris-HCl buffer containing 0.5 M NaCl and 0.5 M Imidazol. The eluated fraction was desalted to 1x EK-buffer (50 mM Tris-HCl, pH 8.0, 10mM CaCl2, 0.1% Tween-20) using a PD-10 column (GE Healthcare), and EK cleavage of the HIS-tag was performed by adding 15 ul EK-max enzyme (Life Technologies) over night (ON) at room temperature (RT).

The cleaved material was diluted 1/5 in 20 mM Tris–HCl pH 7.4 and purified on a 2 ml Q-Sepharose HP column (GE Healthcare), separated with a linear gradient of 15 column volumes of elution buffer (20 mM Tris–HCl, pH 7.4 with 1 M NaCl). The peaks containing the Can f 1-2-4-6 construct (analyzed by SDS-PAGE) were subjected to size exclusion chromatography on a 16/60 Superdex 200 pg column equilibrated in PBS pH 7.4 using ÄKTA Purifier (GE Healthcare), and stored at -80°C until further use. Protein concentration was estimated using the BCA protein assay (Thermo scientific, Chicago, IL, USA) using the BSA standard, and the purity was assessed by SDS-PAGE under reducing conditions.

*SAXS data collection and analysis*

Small angle X-ray scattering data were collected from purified mature Can f 1-2-4-6 solutions on the X33 camera at the EMBL on the DORIS III storage ring (DESY, Hamburg, Germany) . Using a photon counting Pilatus 1M detector at a sample-detector distance of 2.7 m and a wavelength of λ = 1.5 Å, the range of momentum transfer 0.01 < s < 0.6 Å^-1^ was covered (s = 4π sinθ/λ, where 2θ is the scattering angle). Four solute concentrations ranging from 1 to 7 mg/ml were measured. Radiation damage was monitored by comparing eight successive fifteen-second exposures and no significant changes were observed. Data were normalized to the intensity of the transmitted beam and radially averaged. The scattering of the buffer was subtracted and the difference curves were scaled for protein concentration. The low angle data from the lowest concentration were merged with the highest concentration high angle data to yield the final composite scattering curves from which the radius of gyration, the molecular weight and the excluded volume were evaluated by the automated SAXS data analysis pipeline . This curve was used for *ab initio* shape reconstruction with the program GASBOR and for rigid body modelling with the program CORAL .

*Biochemical characterization, preparation of equimolar amounts of allergens and standardization of lipopolysaccharide (LPS) content*

Analytical SEC was performed using ÄKTA Purifier (GE Healthcare) and a Superdex 200 PC 16/60 column, equilibrated in PBS pH 7.4. The secondary structure of purified Can f 1-2-4-6 and an equimolar mix of the four recombinant lipocalins was analyzed by far-UV circular dichroism (CD) spectra as described . Predictions of secondary structure content were made using the DichroWeb on-line server with the SELCON3 and CDSSTR algorithms . Individual molecular weight predictions for the individual lipocalin allergens or the linked vaccine construct were used to create an equimolar mix used in the CD-spectra and subsequent immunological experiments. In all subsequent experiments, single allergens, allergen mixes (designated mix) and the linked vaccine constructs were diluted to be equal to molarity of rCan f 1. The LPS content of all antigens were determined using the limulus amebocyte lysate endochrome assay according to the manufacturer’s instructions (Charles River Endosafe, Charleston, USA). The LPS content were 13, 16, 2 and 4 ng/mg protein for the single allergens Can f 1, Can f 2, Can f 4 and Can f 6, respectively, 11 and 15 ng/mg for the multimer and the mix and 4 and 6 ng/mg for the control allergens Can f 3 and Fel d 4. Splenocytes from control mice, (nCan f 3, n=2 and PBS, n=2) did not proliferate in the presence of any antigen (not shown) suggesting that LPS did not influence the proliferation.

*Statistical analysis*

Pair-wise comparison of allergic subjects for their IgE-reactivity to the accumulated lipocalin allergens with the linked vaccine construct was performed using Wilcoxon matched pairs test. Analysis of splenocyte proliferation data was performed by repeated measurements ANOVA with Tukey’s multiple comparison test. Murine immunoglobulins and blocking ELISA were assessed by Kruskal-Wallis with Dunn’s multiple comparison test, and comparisons between the linked molecule and the equimolar mix were done using the Mann Whitney t-test. All statistical analyses were performed using Graphpad Prism 5.02 software (Graphpad Software Inc., San Diego, CA, USA). P-values < 0.05 were considered significant.

### FIGURE LEGENDS

**Figure S1. Cloning of the Can f 1-2-4-6 construct**. (A) pET28-H6-EK-sgGFP for LIC cloning and Protein Expression in E. coli. (B) Design of the Can f 1-2-4-6 construct. sgGFP – Superglo Green fluorescent protein, H6 – 6x His-tag, EK – enterokinase, LIC – ligase independent cloning site, KanR – Kanamycin resistance

**Figure S2. Antibody responses after immunization with allergens.** (A) Comparison of IgE antibody reactivity to Can f 1-2-4-6 and the mix after immunization with the same antigens in mice. (B) IgG1 and IgG2a-antibodies measured post immunization (graph heading) by coating with rCan f 1, rCan f 2, rCan f 4 or rCan f 6 in ELISA (x-axis). Boxes with median values and horizontal bars denote 50% of values and 1 standard deviation respectively. ** p<0.01, analyzed with Mann Whitney test (A).

**FIGURES**

Figure S1a.


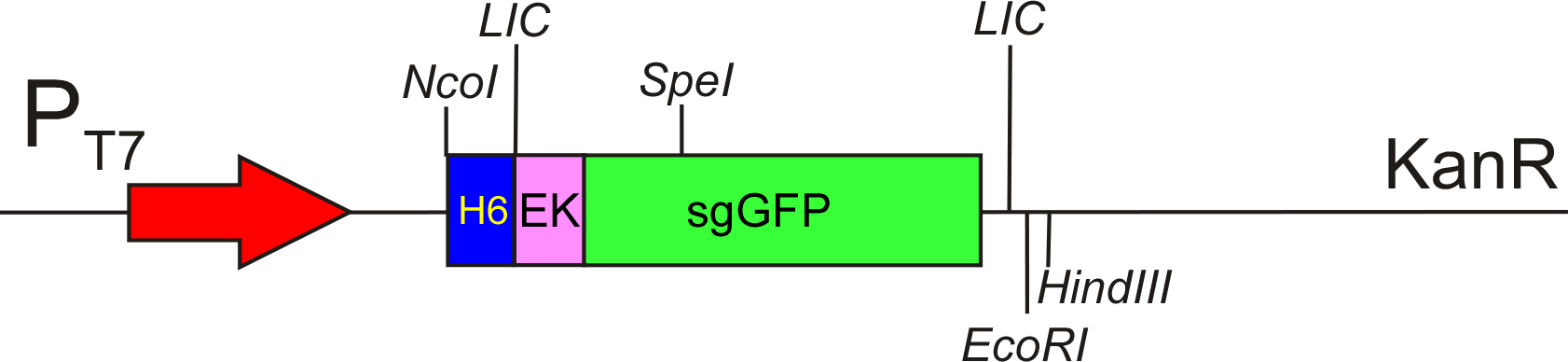


Figure S1b.


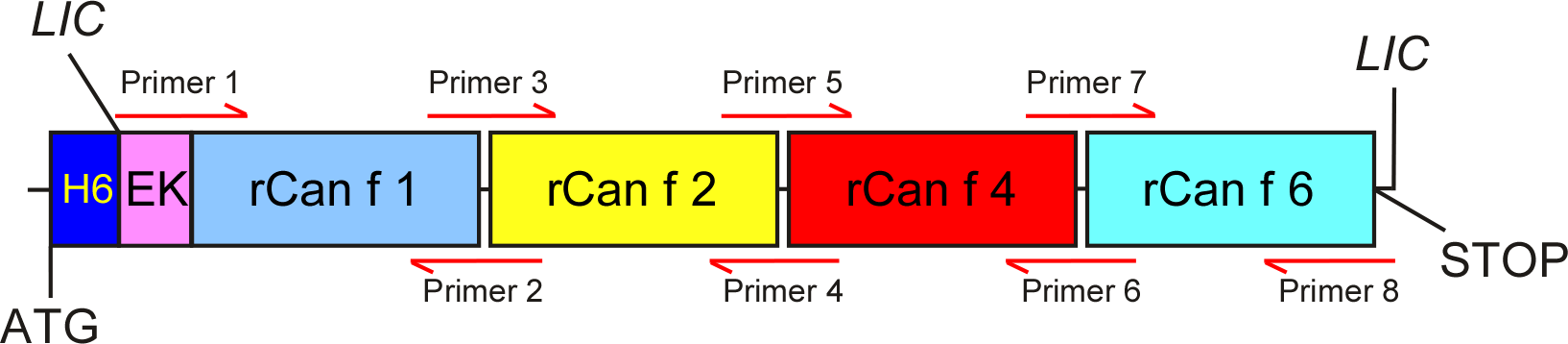


Fig S2a.


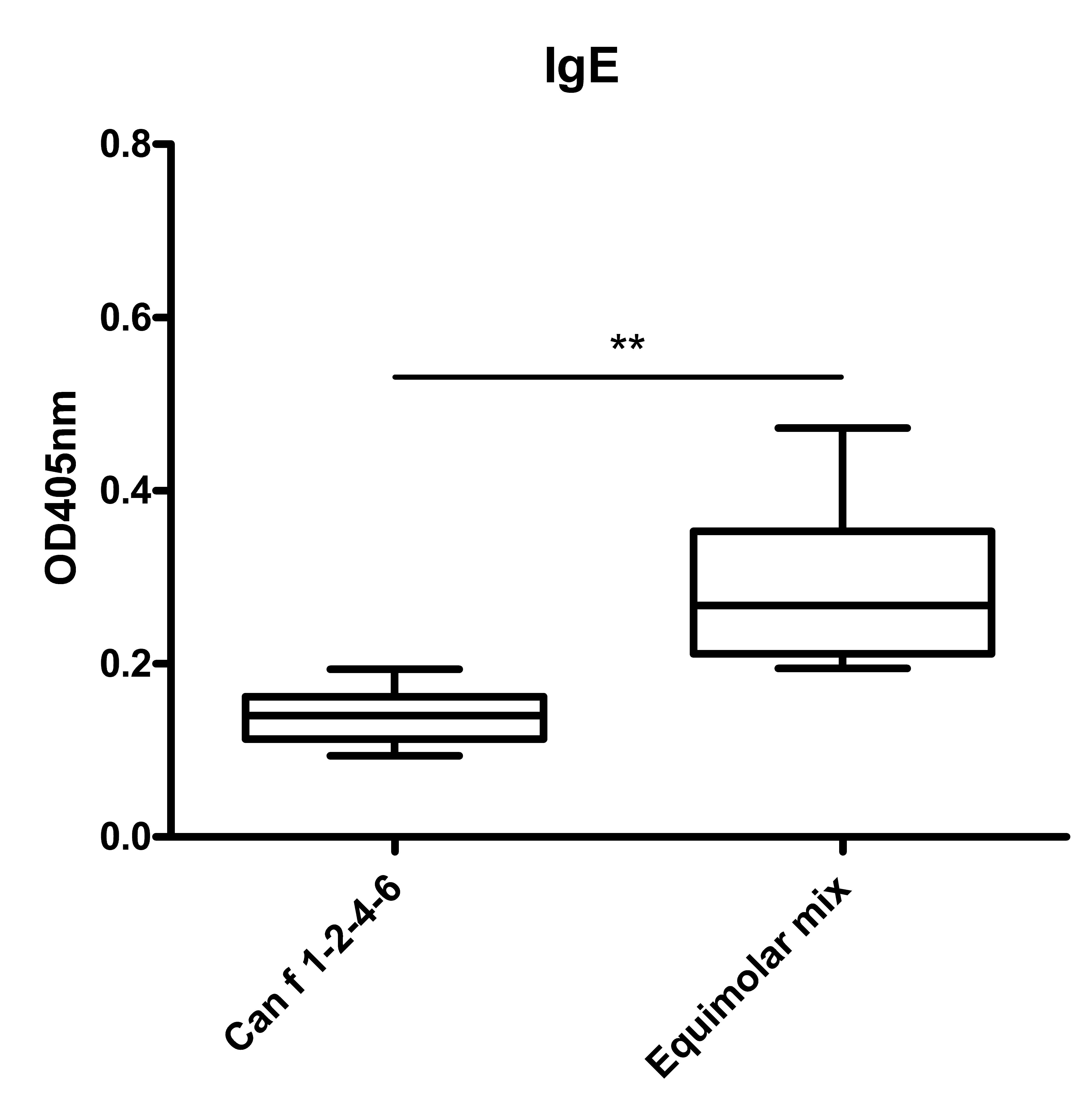


Fig S2b.


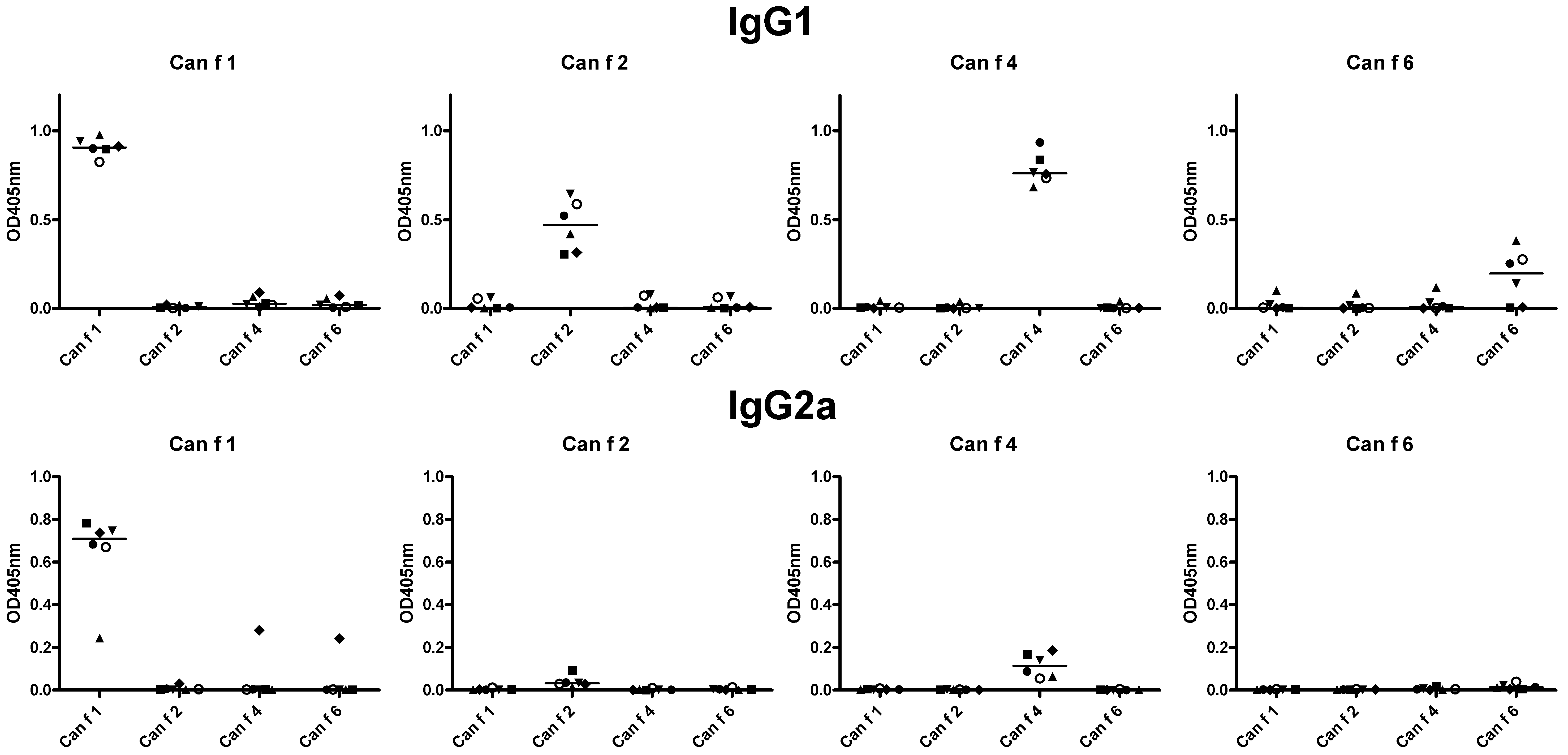


**Table S1.** List of oligonucleotides used for construction of the Can f 1-2-4-6 construct

| ***Primer #*** | | ***Annotation*** | | ***Sequence*** |
| --- | --- | --- | --- | --- |
| ***1*** | ***Can f 1-2-4-6 Fwd*** | | ***TCAGCAAGGGCTGAGGGATCTGTATGATGATGACGATAAAGACACGGTCGCGGTTTCTGGCAAGTGGTACTTA*** | |
| ***2*** | ***Can f 1 Rev*** | | ***ATGGTTACCCTCGCTACCGCTACCCTGTCCTCCTGGAGAGCAGGTTTCGCTCTGTGCTAA*** | |
| ***3*** | ***Can f 2 Fwd*** | | ***CCAGGAGGACAGGGTAGCGGTAGCGAGGGTAACCATGAAGAGCCACAAGGTGGTCTGGAA*** | |
| ***4*** | ***Can f 2 Rev*** | | ***CAGCGGCAGCTGGCTACCGCTACCGTCACGGCTACCTTGACAGCGGTCGTCGTCGCTCAG*** | |
| ***5*** | ***Can f 4 Fwd*** | | ***GGTAGCCGTGACGGTAGCGGTAGCCAGCTGCCGCTGCCGAATGTTCTGACCCAGGTTAGC*** | |
| ***6*** | ***Can f 4 Rev*** | | ***TTCTTCTTCATGGCTACCGCTACCTTCATGATTCGGGCAATTATCGGTATCGCCCAGATA*** | |
| ***7*** | ***Can f 6 Fwd*** | | ***CCGAATCATGAAGGTAGCGGTAGCCATGAAGAAGAAAACGACGTAGTAAAAGGCAATTTT*** | |
| ***8*** | ***Can f 1-2-4-6 Rev*** | | ***TCAGCGGAAGCTGAGGTTATTATTCTGCGCTGCTAACCTGTGCTGCTTCGCTCTGACGTG*** | |
| ***9*** | ***T7 Promoter primer*** | | ***TAATACGACTCACTATAGGG*** | |
| ***10*** | ***T7 Terminator primer*** | | ***GCTAGTTATTGCTCAGCGG*** | |

**Table S2.** SAXS data collection and derived parameters

| Data collection parameters | |
| --- | --- |
| Instrument | EMBL beamline X33 |
| Wavelength (Å) | 1.5 |
| *s* range (Å^-1^) | 0.01–0.6 |
| Exposure time (s) | 15×8 |
| Concentration range (mg/ml) | 1.0–7.0 |
| Temperature (°K) | 283 |
| Structural parameters |  |
| R_g_ (Å) (from Guinier) | 42.1 ± 3.2 |
| R_g_ (Å) (from p(r)) | 44.8 ± 4.5 |
| D_max_ (Å) | 152 ± 15 |
| Molecular mass determination | |
| MW (kDa) from I(0) | 77 ± 8 |
| MM (kDa) from Porod volume | 75 ± 8 |
| Calculated MM (kDa) from sequence | 76.4 |
| Software employed |  |
| Primary data reduction and processing | Automated SAXS data analysis pipeline |
| *Ab initio* reconstruction | GASBOR |
| Rigid body modelling | CORAL |

**Table S3.** Dog sensitized subjects and IgE-levels against tested allergens (Dog dander extract – ImmunoCAP, Individual allergen components – Quantitative IgE ELISA) Data are presented as kU/L (adjusted to each other using reference sera)

| Subject | Dog dander | Can f 1 | Can f 2 | Can f 4 | Can f 6 | Can f 1-2-4-6 | Mix |  | Subject | Dog dander | Can f 1 | Can f 2 | Can f 4 | Can f 6 | Can f 1-2-4-6 | Mix |
| --- | --- | --- | --- | --- | --- | --- | --- | --- | --- | --- | --- | --- | --- | --- | --- | --- |
| 1 | 1,5 | 0 | 0 | 0 | 0 | 0 | 0 |  | 51 | 3,3 | 0 | 0 | 0 | 0 | 0 | 0 |
| 2 | 0,82 | 0,2 | 0 | 0 | 0 | 0,2 | 0 |  | 52 | 8 | 0,1 | 0 | 0 | 0,1 | 0,1 | 0 |
| 3 | 5,9 | 0 | 0 | 0 | 2,2 | 6,4 | 3 |  | 53 | 0,47 | 0 | 0 | 0 | 0 | 0 | 0 |
| 4 | 1,2 | 0 | 0 | 0 | 0 | 0 | 0 |  | 54 | 27 | 6,7 | 2,4 | 7,4 | 4,5 | 17 | 12,3 |
| 5 | 1,1 | 0 | 0 | 0 | 0 | 0 | 0 |  | 55 | 1,4 | 0 | 0 | 0 | 0 | 0 | 0 |
| 6 | 0,77 | 0 | 0 | 0 | 0 | 0 | 0 |  | 56 | 1,1 | 0 | 0 | 0 | 0 | 0,2 | 0 |
| 7 | 2,2 | 0 | 0 | 0 | 0 | 0 | 0 |  | 57 | 1,1 | 0,2 | 0 | 0 | 0 | 0,2 | 0,1 |
| 8 | >100 | 220 | 175 | 103 | 191 | 900 | 316 |  | 58 | 2,4 | 0 | 0 | 0 | 0 | 0 | 0 |
| 9 | 3,1 | 0 | 0 | 0 | 0 | 0,1 | 0 |  | 59 | 2,2 | 0 | 0 | 0 | 1,4 | 3,7 | 0,2 |
| 10 | 0,61 | 0 | 0 | 0,1 | 0 | 0,1 | 0 |  | 60 | 0,68 | 0 | 0 | 0 | 0 | 0 | 0 |
| 11 | 3,6 | 0,2 | 0 | 0 | 0 | 0,7 | 0,3 |  | 61 | 7,6 | 2 | 0,1 | 0 | 1,7 | 6,7 | 3,7 |
| 12 | 10 | 0 | 0 | 0 | 0 | 0 | 0 |  | 62 | 0,9 | 0,2 | 0 | 0 | 0,2 | 0,8 | 0,2 |
| 13 | 0,93 | 0 | 0 | 0 | 0 | 0 | 0 |  | 63 | 0,75 | 0 | 0 | 0 | 0 | 0 | 0 |
| 14 | 0,71 | 0 | 0 | 0 | 0,2 | 0,8 | 0,1 |  | 64 | 7,7 | 1,9 | 0 | 0 | 2,6 | 8,1 | 3,3 |
| 15 | 0,36 | 0 | 0 | 0 | 0 | 0 | 0 |  | 65 | 6 | 0,1 | 0,2 | 0 | 1 | 2,5 | 1,2 |
| 16 | 3 | 1,1 | 0 | 0 | 0 | 1,5 | 0,8 |  | 66 | 13 | 0,2 | 0,2 | 2,7 | 2 | 6,1 | 4,2 |
| 17 | 6,3 | 0 | 0 | 0 | 0,6 | 1,4 | 1 |  | 67 | 4,6 | 1,9 | 0 | 0 | 0 | 3,1 | 1,9 |
| 18 | 0,36 | 0 | 0 | 0 | 0 | 0 | 0 |  | 68 | 1,3 | 0 | 0,1 | 0 | 0,1 | 0,5 | 0,2 |
| 19 | 0,7 | 0 | 0 | 0 | 0 | 0 | 0 |  | 69 | 3,4 | 0 | 0 | 0 | 0,1 | 0,2 | 0,1 |
| 20 | 1,2 | 1,5 | 0 | 0 | 0,1 | 2 | 1,3 |  | 70 | 44 | 0 | 0 | 0 | 68 | 129 | 47,9 |
| 21 | 18 | 10,2 | 4,3 | 7,1 | 12,4 | 77,1 | 28,3 |  | 71 | 2,1 | 0,2 | 0 | 0 | 0 | 0,5 | 0,2 |
| 22 | 0,92 | 0 | 0 | 0 | 0 | 0 | 0 |  | 72 | 1,5 | 0 | 0 | 0 | 0 | 0 | 0 |
| 23 | 0,87 | 0,3 | 0 | 0 | 0 | 0,5 | 0,3 |  | 73 | 0,64 | 0 | 0 | 0 | 0,1 | 0,3 | 0,1 |
| 24 | 12 | 19,3 | 0 | 0 | 0 | 27,6 | 12,1 |  | 74 | 10 | 0,9 | 1,9 | 0 | 0,1 | 4,7 | 2,4 |
| 25 | 0,49 | 0,2 | 0 | 0 | 0 | 0,5 | 0,2 |  | 75 | 0,35 | 0 | 0 | 0 | 0 | 0 | 0 |
| 26 | 1,7 | 0 | 0 | 0 | 0 | 0 | 0 |  | 76 | 0,42 | 0 | 0 | 0 | 0 | 0 | 0 |
| 27 | 18 | 0 | 0 | 0 | 0 | 0 | 0 |  | 77 | 6,4 | 0 | 0 | 0 | 0 | 0 | 0 |
| 28 | 2 | 0 | 0 | 0 | 0 | 0 | 0 |  | 78 | 24 | 0 | 0 | 0 | 0 | 0 | 0 |
| 29 | 2,4 | 0,1 | 0 | 0 | 0 | 0,1 | 0,1 |  | 79 | 33 | 4,2 | 0 | 4,9 | 2,6 | 22,1 | 12,6 |
| 30 | 0,66 | 0 | 0 | 0 | 0 | 0 | 0 |  | 80 | 6,3 | 0,3 | 0 | 5,7 | 6,3 | 17,4 | 9,8 |
| 31 | 33 | 27,5 | 10,4 | 0,1 | 0,1 | 45,3 | 46,2 |  | 81 | 0,61 | 0 | 0 | 0 | 0 | 0 | 0 |
| 32 | 4,3 | 0 | 0 | 0 | 0 | 0 | 0 |  | 82 | >100 | 177 | 67 | 0 | 216 | 1072 | 468 |
| 33 | 18 | 8,4 | 1,2 | 6,1 | 3,5 | 34 | 18,8 |  | 83 | 35 | 41 | 0,1 | 0 | 0,2 | 79 | 44,7 |
| 34 | 2,9 | 0,6 | 0 | 0 | 20,8 | 24,5 | 13,8 |  | 84 | 11 | 0,5 | 0 | 0,2 | 0 | 3,8 | 0,6 |
| 35 | 0,94 | 0 | 0 | 0 | 0 | 0 | 0 |  | 85 | 18 | 12,6 | 0 | 0 | 12,5 | 75 | 20,4 |
| 36 | 4 | 0,1 | 0,3 | 0 | 0,2 | 0,2 | 0,1 |  | 86 | 0,58 | 0 | 0 | 0 | 0 | 0 | 0 |
| 37 | 5,6 | 1,2 | 0 | 0 | 0,2 | 1,8 | 1,2 |  | 87 | 1,8 | 0 | 0 | 0 | 0 | 0 | 0 |
| 38 | 3,5 | 1,2 | 0 | 0 | 4,6 | 6,8 | 3,4 |  | 88 | 1,1 | 0 | 0 | 0 | 0 | 0 | 0 |
| 39 | 0,35 | 0 | 0 | 0 | 0 | 0 | 0 |  | 89 | 1,5 | 0 | 0 | 0 | 0 | 0 | 0 |
| 40 | 3,2 | 0 | 0 | 0 | 0 | 0 | 0 |  | 90 | 25 | 0 | 0 | 0 | 0 | 0 | 0 |
| 41 | 0,47 | 0 | 0 | 0 | 0 | 0 | 0 |  | 91 | 6,3 | 7,6 | 0 | 0 | 1,3 | 10,9 | 8,7 |
| 42 | 3,6 | 0,3 | 0,3 | 0,4 | 0,5 | 0,8 | 0,4 |  | 92 | 4,1 | 0,4 | 0 | 0 | 0 | 0,7 | 0,4 |
| 43 | 1,2 | 0,5 | 0 | 0 | 0 | 1 | 0,4 |  | 93 | 9,7 | 0 | 0 | 0 | 0 | 0 | 0 |
| 44 | 1,7 | 0,9 | 0 | 0 | 0,1 | 2,2 | 1 |  | 94 | 2,1 | 0,7 | 0 | 0 | 0,1 | 1,8 | 0,8 |
| 45 | 22 | 13,8 | 0 | 0 | 3 | 19,4 | 8,9 |  | 95 | 0,81 | 0 | 0 | 0,5 | 0 | 1,4 | 0,5 |
| 46 | 28 | 22,3 | 0 | 0 | 27,8 | 123 | 34,3 |  | 96 | 2,3 | 0 | 0 | 0 | 0 | 0,1 | 0 |
| 47 | 18 | 24,3 | 0 | 0 | 1,1 | 34,8 | 13 |  | 97 | 3,2 | 0,1 | 0,1 | 0 | 0 | 0,1 | 0,1 |
| 48 | 0,81 | 0 | 0 | 0 | 0 | 0 | 0 |  | 98 | 1,5 | 0 | 0 | 0 | 0 | 0 | 0 |
| 49 | 1,8 | 0 | 0 | 0,1 | 0 | 0,1 | 0,1 |  | 99 | 2,4 | 1,8 | 0 | 0,5 | 1,5 | 7,3 | 4,1 |
| 50 | 0,65 | 0 | 0 | 0 | 0 | 0 | 0 |  | 100 | 7,8 | 0,1 | 0,2 | 0 | 0,2 | 0,2 | 0,2 |

### References

1. Madhurantakam C, Nilsson OB, Uchtenhagen H, Konradsen J, Saarne T, et al. (2010) Crystal structure of the dog lipocalin allergen Can f 2: implications for cross-reactivity to the cat allergen Fel d 4. J Mol Biol 401: 68-83.

2. Nilsson OB, Binnmyr J, Zoltowska A, Saarne T, van Hage M, et al. (2012) Characterization of the dog lipocalin allergen Can f 6: the role in cross-reactivity with cat and horse. Allergy 67: 751-757.

3. Blanchet CE, Zozulya AV, Kikhney AG, Franke D, Konarev PV, et al. (2012) Instrumental setup for high-throughput small- and wide-angle solution scattering at the X33 beamline of EMBL Hamburg. Journal of Applied Crystallography 45: 489-495.

4. Franke D, Kikhney AG, Svergun DI (2012) Automated acquisition and analysis of small angle X-ray scattering data. Nuclear Instruments & Methods in Physics Research Section a-Accelerators Spectrometers Detectors and Associated Equipment 689: 52-59.

5. Svergun DI, Petoukhov MV, Koch MHJ (2001) Determination of domain structure of proteins from X-ray solution scattering. Biophysical Journal 80: 2946-2953.

6. Petoukhov MV, Franke D, Shkumatov AV, Tria G, Kikhney AG, et al. (2012) New developments in the ATSAS program package for small-angle scattering data analysis. Journal of Applied Crystallography 45: 342-350.

7. Whitmore L, Wallace BA (2008) Protein secondary structure analyses from circular dichroism spectroscopy: methods and reference databases. Biopolymers 89: 392-400.

8. Sreerama N, Woody RW (1993) A self-consistent method for the analysis of protein secondary structure from circular dichroism. Anal Biochem 209: 32-44.

9. Johnson WC (1999) Analyzing protein circular dichroism spectra for accurate secondary structures. Proteins 35: 307-312.
